# Supplementary figures and images for: Maturation and detoxification of synphilin-1 inclusion bodies regulated by sphingolipids
Source: eLife. 2025 Feb 10;12:RP92180. doi: 10.7554/eLife.92180 (PMC11810108; doi:10.7554/eLife.92180)

**Fig. 4D**

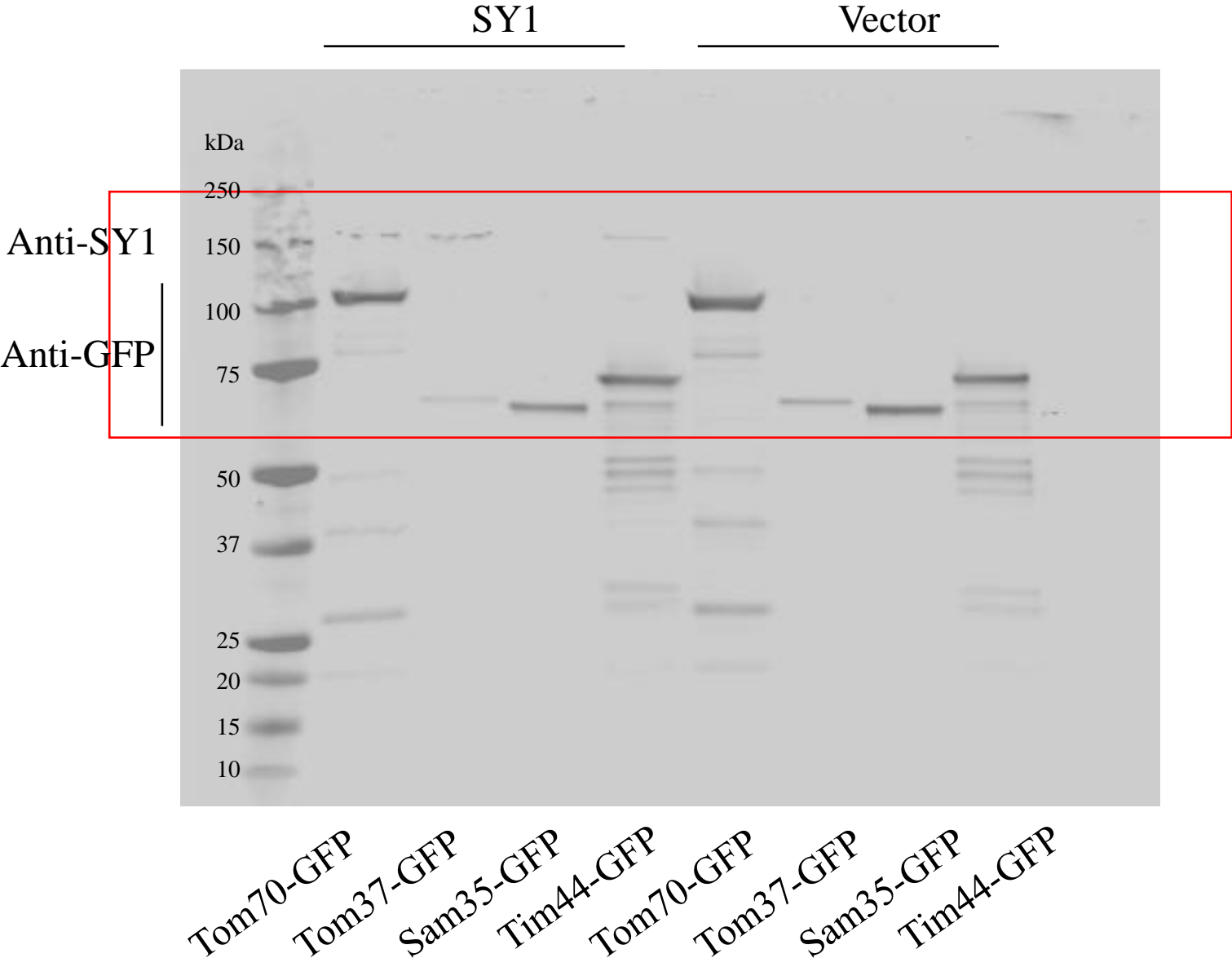

Supplement: Figure 4—source data 1. [file elife-92180-fig4-data1.pdf]

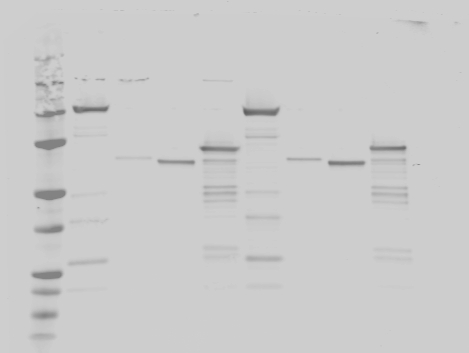

Supplement: Figure 4—source data 2. [file elife-92180-fig4-data2.zip › Tom70 37 Sam35 Tim44 SY1.jpg]

Figure 5-figure supplement 1 A

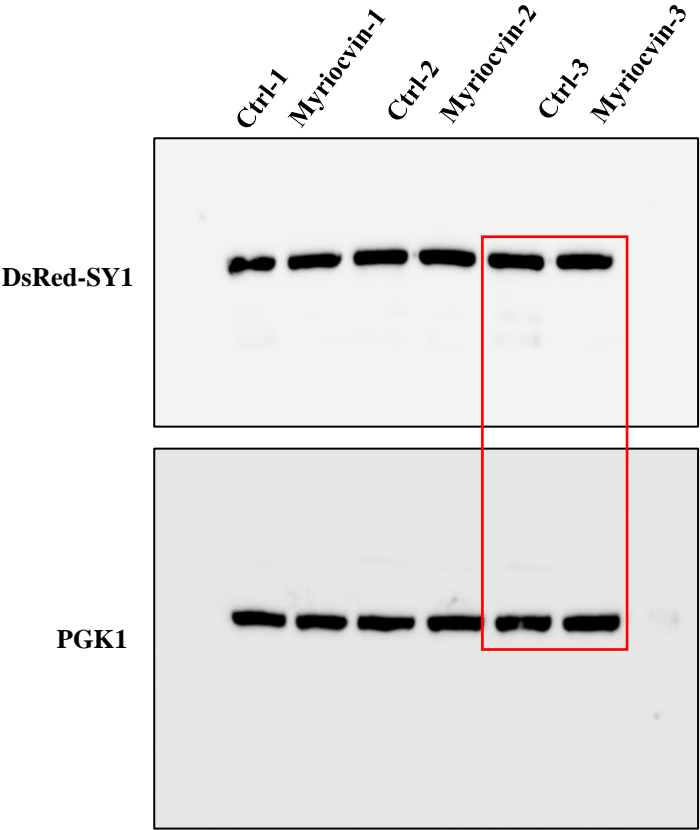

Figure 5-figure supplement 1 B

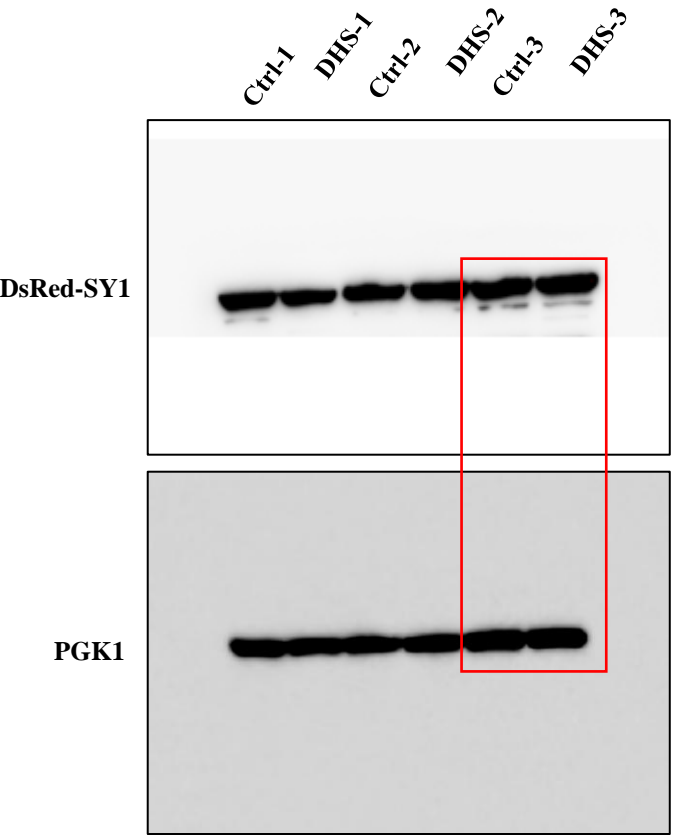

Supplement: Figure 5—figure supplement 1—source data 1. [file elife-92180-fig5-figsupp1-data1.pdf]

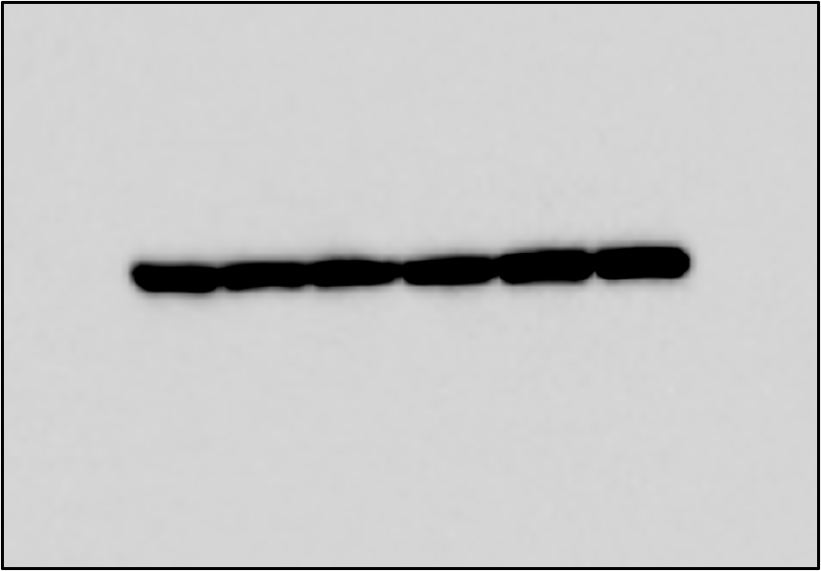

Supplement: Figure 5—figure supplement 1—source data 2. [file elife-92180-fig5-figsupp1-data2.zip › PGK1 DHS.tif]

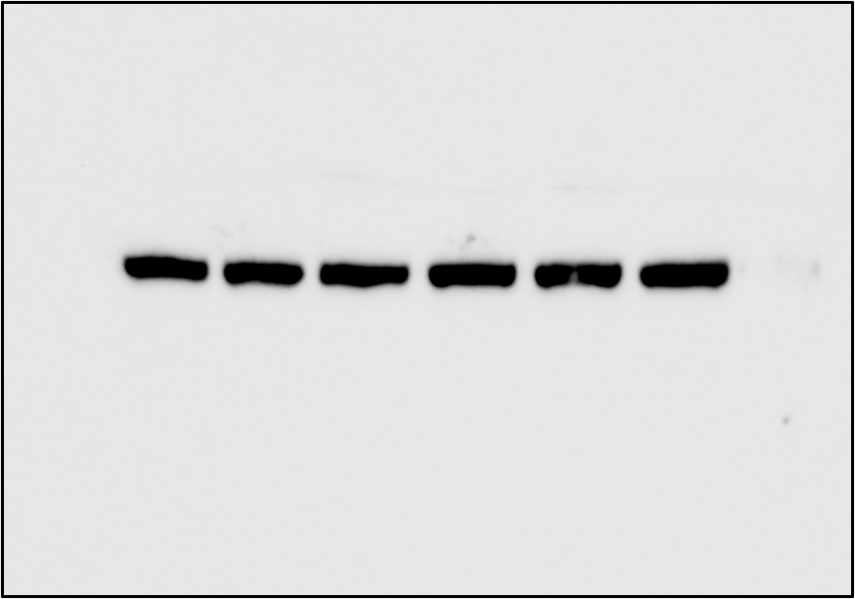

Supplement: Figure 5—figure supplement 1—source data 2. [file elife-92180-fig5-figsupp1-data2.zip › PGK1 Myriocin.tif]

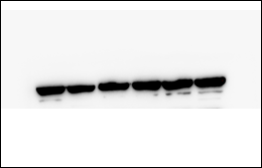

Supplement: Figure 5—figure supplement 1—source data 2. [file elife-92180-fig5-figsupp1-data2.zip › SY1 DHS.tif]

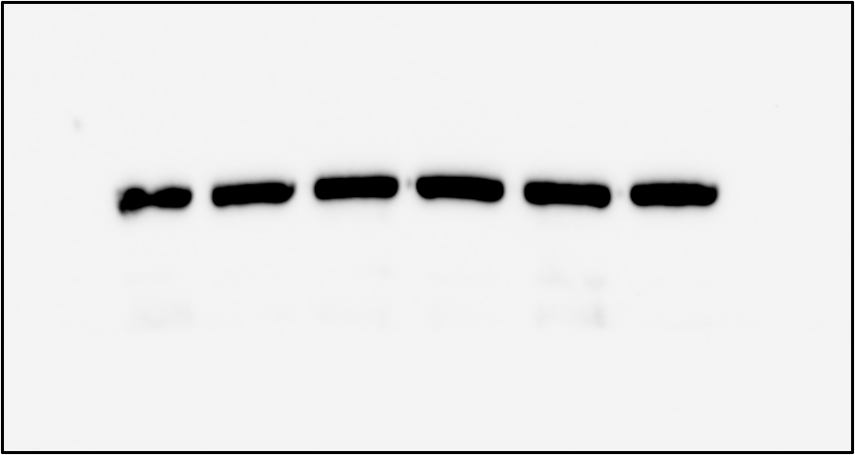

Supplement: Figure 5—figure supplement 1—source data 2. [file elife-92180-fig5-figsupp1-data2.zip › SY1 Myriocin.tif]

### Figure 6-figure supplement 3

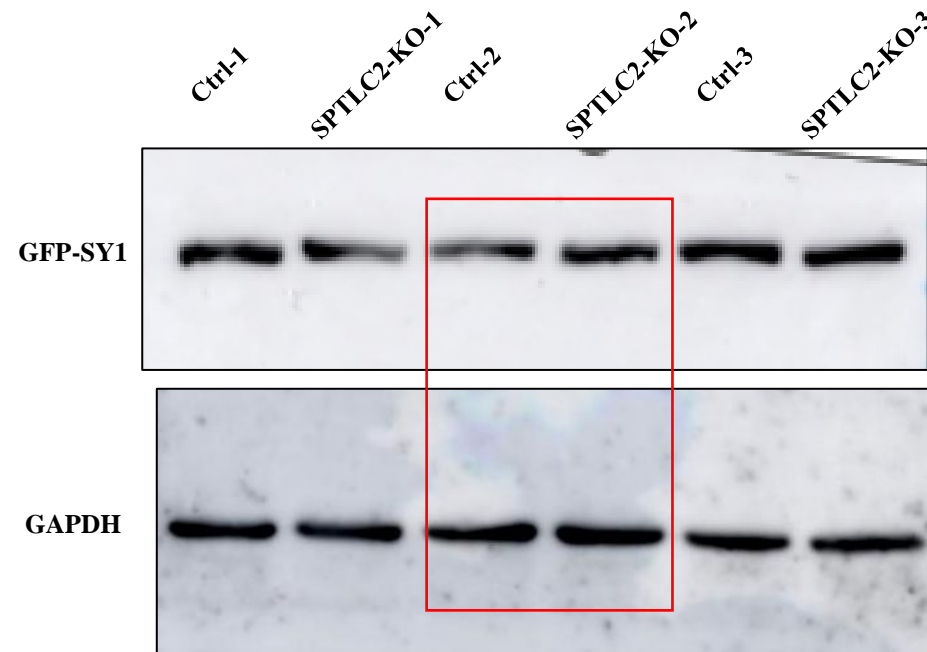

Supplement: Figure 6—figure supplement 3—source data 1. [file elife-92180-fig6-figsupp3-data1.zip › Figure 6XXXfigure supplement 3XXXsource data 1.pdf]

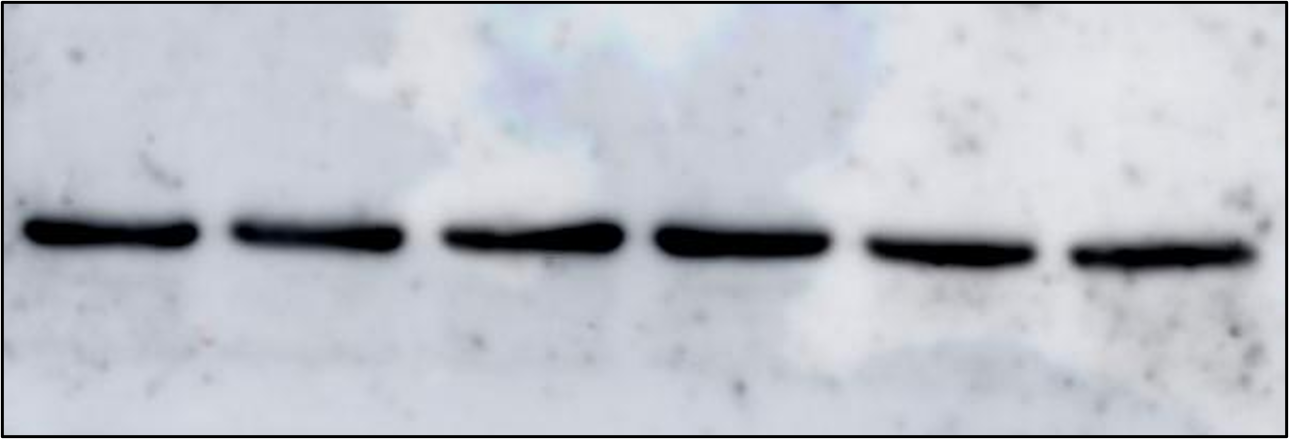

Supplement: Figure 6—figure supplement 3—source data 2. [file elife-92180-fig6-figsupp3-data2.zip › GAPDH Ctrl and SPTLC2-KO.tif]

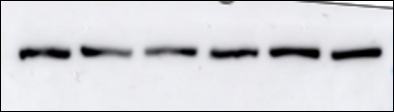

Supplement: Figure 6—figure supplement 3—source data 2. [file elife-92180-fig6-figsupp3-data2.zip › SY1 Ctrl and SPTLC2-KO.tif]

Figure 7-figure supplement 1 B

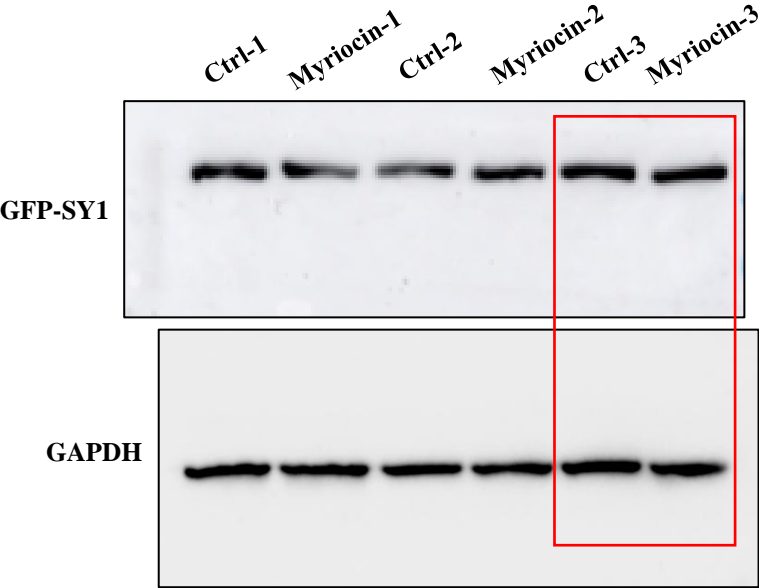

Figure 7-figure supplement 1 C

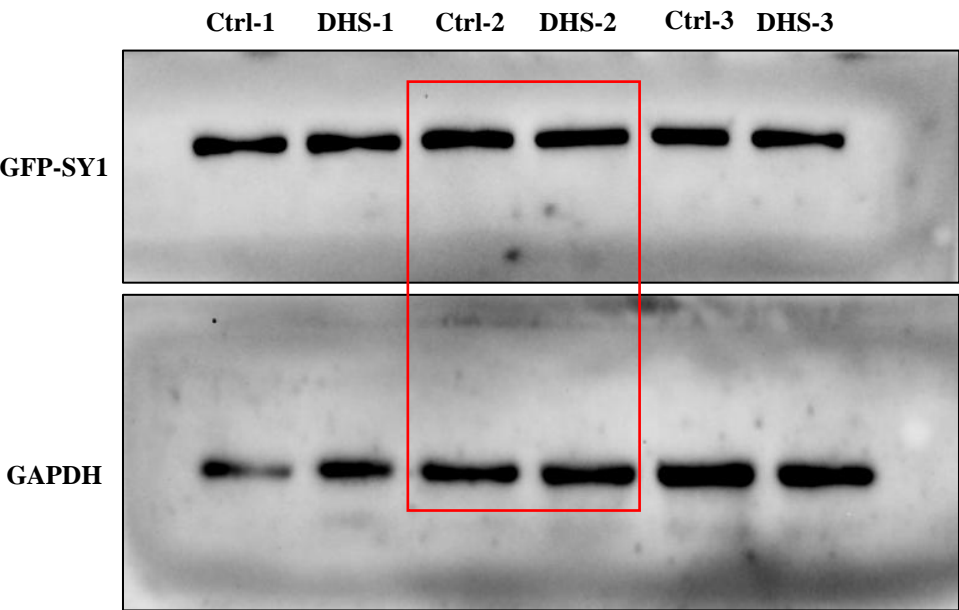

Figure 7-figure supplement 1 D

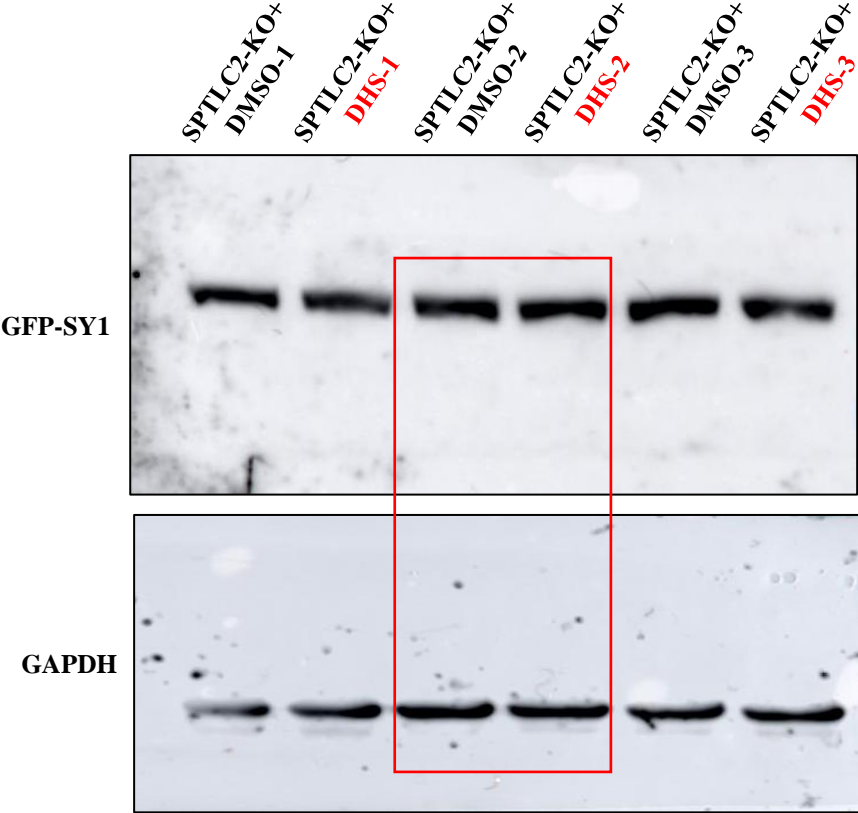

Supplement: Figure 7—figure supplement 1—source data 1. [file elife-92180-fig7-figsupp1-data1.pdf]

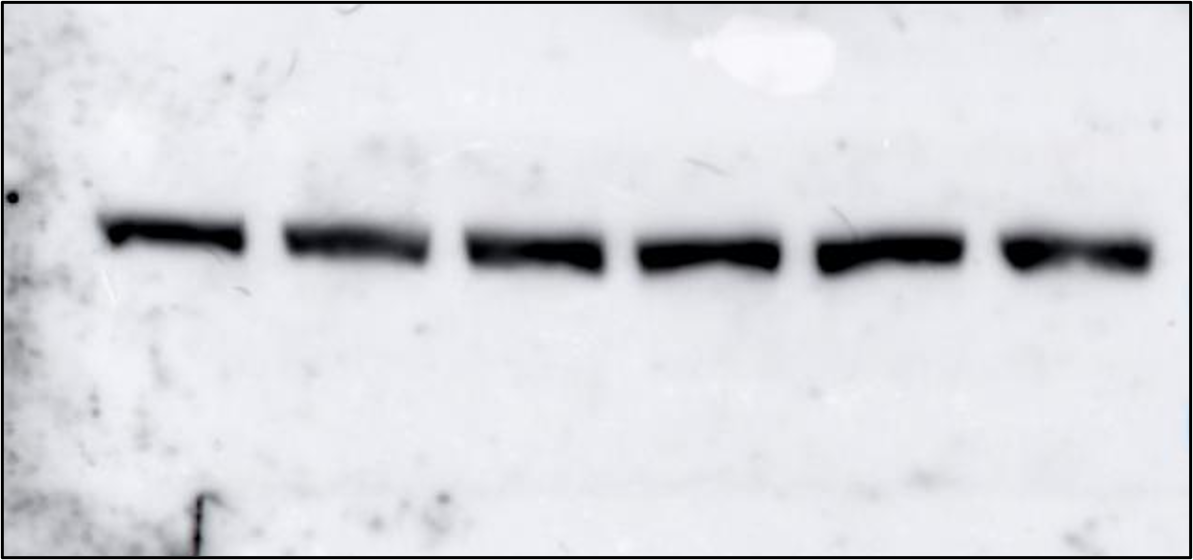

Supplement: Figure 7—figure supplement 1—source data 2. [file elife-92180-fig7-figsupp1-data2.zip › SY1 SPTLC2-KO DHS.tif]

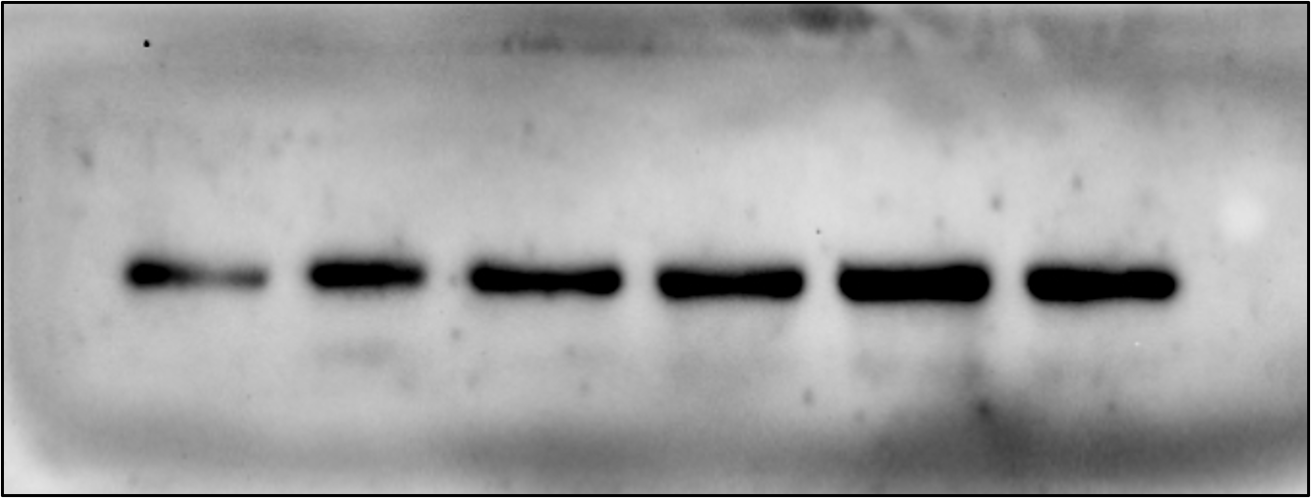

Supplement: Figure 7—figure supplement 1—source data 2. [file elife-92180-fig7-figsupp1-data2.zip › GAPDH DHS.tif]

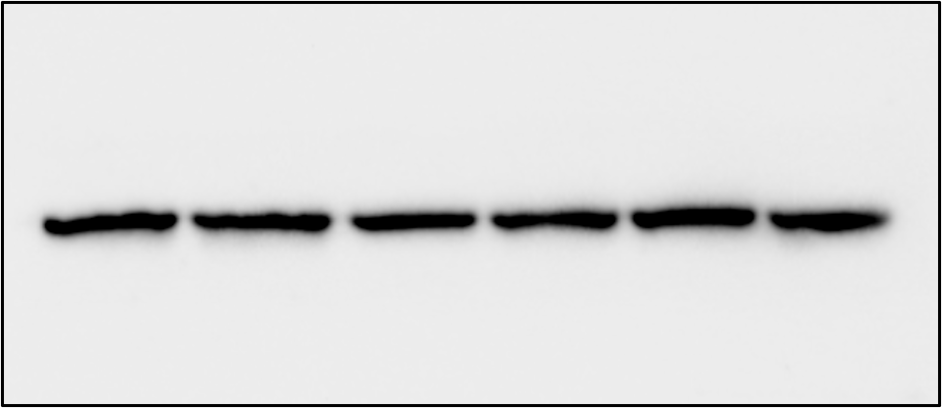

Supplement: Figure 7—figure supplement 1—source data 2. [file elife-92180-fig7-figsupp1-data2.zip › GAPDH Myriocin.tif]

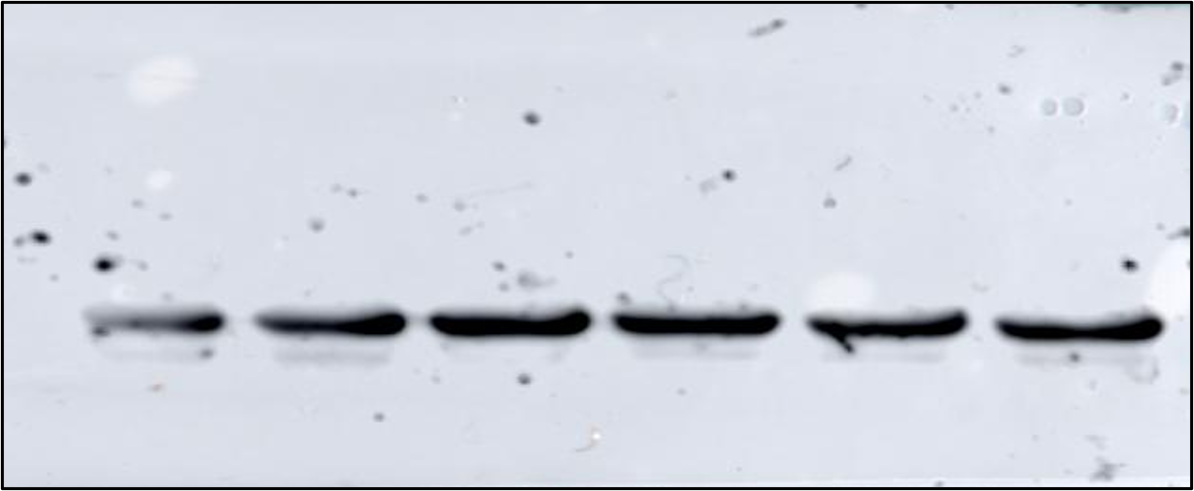

Supplement: Figure 7—figure supplement 1—source data 2. [file elife-92180-fig7-figsupp1-data2.zip › GAPDH SPTLC2-KO DHS.tif]

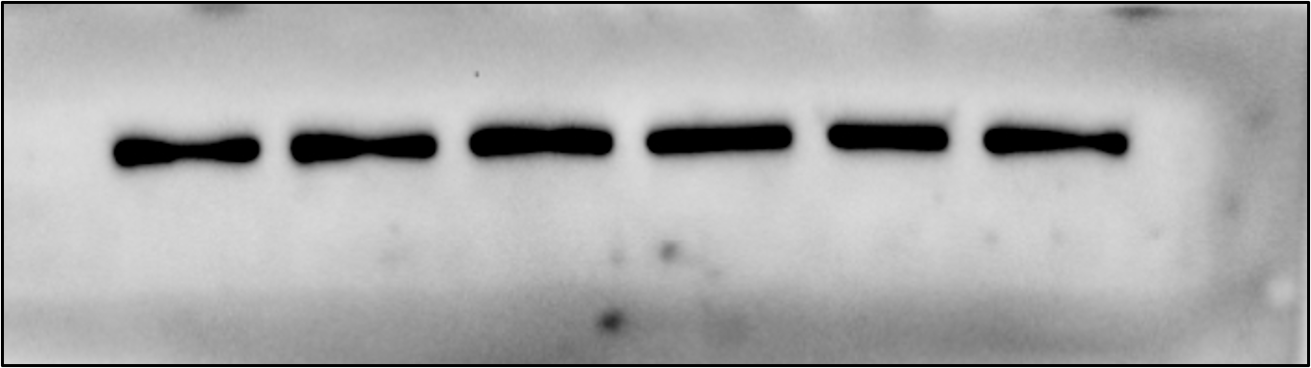

Supplement: Figure 7—figure supplement 1—source data 2. [file elife-92180-fig7-figsupp1-data2.zip › SY1 DHS.tif]

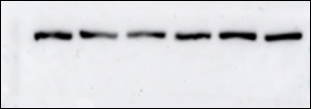

Supplement: Figure 7—figure supplement 1—source data 2. [file elife-92180-fig7-figsupp1-data2.zip › SY1 Myriocin.tif]
